# Formation of Twin Boundaries in Rapidly Solidified Metals through Deformation Twinning

**DOI:** 10.3390/ma16134503

**Published:** 2023-06-21

**Authors:** Binting Huang, Jishi Yang, Zhiheng Luo, Yang Wang, Nan Wang

**Affiliations:** 1 Department of Materials Science and Engineering, Guangdong Technion—Israel Institute of Technology, Shantou 515063, China; huang.binting@gtiit.edu.cn (B.H.);; 2 Guangdong Provincial Key Laboratory of Materials and Technologies for Energy Conversion, Guangdong Technion—Israel Institute of Technology, Shantou 515063, China; 3 State Key Laboratory of Rolling and Automation, Shenyang 110819, China; 4 Department of Materials Science and Engineering, Technion—Israel Institute of Technology, Haifa 3200003, Israel

**Keywords:** twinning, solidification, phase field

## Abstract

The rapid solidification process is relevant to many emerging metallurgical technologies. Compared with conventional solidification processes, high-density microstructure defects and residual thermal stress are commonly seen in rapidly solidified metals. Among the various defects, potentially beneficial twin boundaries have been observed in the rapidly solidified nanocrystalline microstructures of many alloy systems. In this work, a pathway for forming twin boundaries in rapid solidification processes is proposed. A detailed derivation of strain inhomogeneities upon thermal shrinkage and the deformation twinning phase field method is given. By calculating cooling-induced thermal strain inhomogeneity in nanocrystalline metals and growth thresholds for deformation twinning using the phase field method, it is shown that residual thermal strain hotspots in the microstructure can reach the threshold for deformation twinning when the shear elastic property of grain boundaries is significantly different from the bulk.

## 1. Introduction

Rapid solidification technologies for metals have been under development for several decades. Depending on the cooling rate, the solidified metal may form nanocrystalline structures or amorphous metallic glasses. While nanocrystalline metals have been known for their high strength due to blockage of dislocation motions by high-density grain boundaries (GB), their relatively low ductility may pose an issue in applications that require failure resistance [1]. Twin boundaries have been used extensively in grain-boundary engineering to improve the mechanical properties of polycrystalline materials, but it is only recently observed that, through rapid solidification processes, a significant fraction of twin boundaries may be introduced into the metal microstructures [2,3].

Growth twinning and deformation twinning are the two classical mechanisms of twin formation in metals. It is proposed that twin boundaries could be formed through recrystallization and growth twinning in rapid solidification processes. Through the quench of the Ni80Cu20 alloy, followed by subsequent annealing, Xu et al. observed the formation of twin boundaries in their study [3]. Phma et al. used transmission electron microscopy to reveal the presence of deformation nanotwins, which were formed due to the high-strain rapid solidification inherent to laser powder bed fusion of austenitic stainless steels [4]. Wang et al. subjected a Ni-Cu alloy to high undercooling and observed the presence of high-angle grain boundaries and twins, indicating the occurrence of recrystallization under rapid solidification [5]. However, due to the interactions of many important factors involved in the rapid solidification processes, including microstructures, stresses, liquid transport, etc., a detailed picture of the formation of twin boundaries in rapidly solidified microstructures is still not fully clarified.

In this work, a twin boundary formation mechanism involving residual thermal stresses and the deformation twinning in rapidly solidified microstructures is proposed. By calculating thermally induced strain inhomogeneity in nanocrystalline metals [6] and the critical strain for deformation twin growth using a phase-field (PF) method [7], it is shown that the large temperature drop in rapid solidification processes can create strain hotspots in the solidified nanocrystalline structure within which deformation twinning may happen. This previously unexplored thermal shrinkage-induced deformation twinning mechanism may provide an alternative pathway for the formation of twin structures in rapidly solidified metals.

## 2. Materials and Methods

### 2.1. Thermal Strain Inhomogeneity in Polycrystalline Structures

Understanding the elastic response of materials microstructure under external deformation is an essential part of the study of the mechanical properties of materials. The microstructure is not only a heterogeneous elastic media with complex structures, it also evolves and changes its properties under the elastic load. During the structural evolution, interfaces between different parts of the microstructure may change from the coherent state to the semi-coherent or incoherent states and vastly modify the elastic strain field on both sides of the interface. To apply the continuum elasticity theory to microstructures, a key parameter is the elastic property of the microstructural interfaces [7]. For the nanocrystalline structure formed in rapid solidification processes, the elastic property of GBs is a critical parameter. However, despite its significance, our knowledge of the elastic properties of various grain boundaries in nanocrystalline structure is still very limited. Experimental studies have shown that both Young’s modulus and the shear modulus decrease with average grain size in nanocrystalline metals [8,9]. A previous numerical study based on continuum elasticity theory also predicted a smaller Young’s modulus for nanocrystalline materials [10]. By treating the GB as a region with a smaller density, Fecht et al. [11] found that the bulk modulus of the GB gradually decreases to zero when the GB atomic density is about 50% of the bulk value under hydrostatic compressive pressure. The presence of voids and precipitates at the GB may also significantly modify the elastic response of the boundary [12].

Pre-melting of the grain boundaries at a high temperature is another mechanism that may drastically change the elastic shear response of the polycrystalline materials. Many simulations and experimental results [13,14,15,16,17,18] have shown that a quasi-liquid layer may form at the GB when the temperature is below the bulk melting temperature $T_{m}$, the thickness of this quasi-liquid layer depends on the misorientation of the GB and it diverges as the temperature approaches the $T_{m}$. Broughton et al. [19] examined the resistance to shear of a (310) symmetric tilt boundary using molecular dynamics. The results suggested that the resistance to sliding at the GB vanishes in the presence of interface pre-melting.

To account for the various factors that may contribute to the elastic properties of the GB in the calculation of thermally induced strain inhomogeneity in the nanocrystalline structure, the following assumptions were used: 1. The GB elastic properties are assumed to be independent of temperature. 2. In order to account for the potential drastic change of the GB properties discussed above, the strain inhomogeneity was calculated and compared using various fractions of the bulk shear modulus for all materials discussed in this work. 3. The elastic properties in the grain interiors are considered independent of the grain lattice orientation. While the approximations introduced above are strong, the calculation results should be sufficient to give, at least, a semi-quantitative picture of the strain inhomogeneity in the nanocrystalline structure.

**Figure 1:**
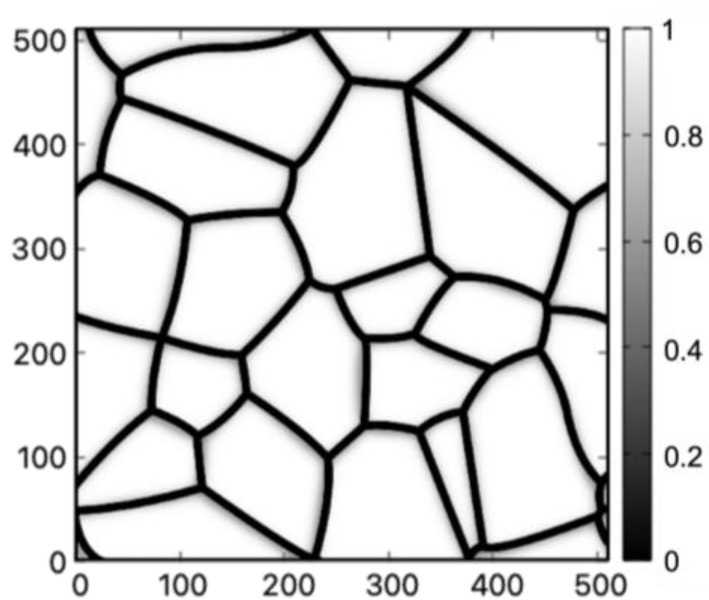
Grain structure from the PF method. The grain interiors are colored in white while the boundaries are colored based on the color bar to the right where the coloring values are obtained following the method given in Ref. [20]. The tics along the x and y axes are shown in arbitrary units.

A polycrystalline grain structure, as shown in Figure 1, is obtained using a previously developed PF method [20].

While the material is undergoing a volumetric shrinkage from the cooling process, strain inhomogeneities will develop in the grain structure due to the difference in elastic properties between the bulk and the GBs. Following the method introduced by Hu et al. [21] and Saswata Bhattacharyya et al. [6], the local strain distribution, $\varepsilon_{ij}(r)$, can be obtained by solving the mechanical equilibrium equation by assuming linear elasticity:

(1) $$\frac{\partial \sigma_{ij}\left(r\right)}{\partial r_{j}}=0,$$

where r is the spatial coordinates, $\sigma_{ij}\left(r\right)=C_{ijkl}\left(r\right)\varepsilon_{kl}(r)$ is the local stress tensor, and $C_{ijkl}\left(r\right)$ is the elastic stiffness tensor which varies from the grain interiors to the GBs. The total strain of the system $\varepsilon_{ij}\left(r\right)$ can be separated into a homogeneous strain ${\bar{\varepsilon }}_{ij}$ and a position-dependent heterogeneous strain $\delta \varepsilon_{ij}(r)$ is as follows

(2) $$\varepsilon_{ij}\left(r\right)={\bar{\varepsilon }}_{ij}+\delta \varepsilon_{ij}\left(r\right),$$

where the homogeneous strain ${\bar{\varepsilon }}_{ij}$ comes from the thermal shrinkage, and the heterogeneous strain $\delta \varepsilon_{ij}(r)$ expresses the spatial-dependent deformation caused by the stiffness difference in the microstructure after the thermal shrinkage. The spatial-dependent elastic stiffness tensor $C_{ijkl}\left(r\right)$ is a sum of a homogeneous tensor $C_{ijkl}^{0}$ and an inhomogeneous perturbation part $\delta C_{ijkl}(r)$

(3) $$C_{ijkl}\left(r\right)=C_{ijkl}^{0}+\delta C_{ijkl}\left(r\right)$$

where the homogenous part is assigned the value of the grain interiors and the perturbation part is used to incorporate the deviation of the elastic properties of the GBs from the grain interiors.

Using Equations (2) and (3), the mechanical equilibrium condition in Equation (1) is written as

(4) $$\frac{\partial \sigma_{ij}\left(r\right)}{\partial r_{j}}={𝛻}_{j}\left[C_{ijkl}^{0}+\delta C_{ijkl}\left(r\right)\right]\left[{\bar{\varepsilon }}_{kl}+\delta \varepsilon_{kl}\left(r\right)\right]=0,$$

where ${𝛻}_{j}=\frac{\partial }{\partial r_{j}}$.

Equation (4) above can be rearranged in the following form

(5) $${𝛻}_{j}\left[C_{ijkl}^{0}{\bar{\varepsilon }}_{kl}+C_{ijkl}^{0}{\delta \varepsilon }_{kl}\left(r\right)+\delta C_{ijkl}\left(r\right){\bar{\varepsilon }}_{kl}+\delta C_{ijkl}\left(r\right)\delta \varepsilon_{kl}\left(r\right)\right]=0.$$

In the first-order approximation, the term $\delta C_{ij}\left(r\right)\delta \varepsilon_{kl}\left(r\right)$ in the equation above can be neglected since it is a product of the two perturbation terms. Since the derivative of $C_{ijkl}^{0}{\bar{\varepsilon }}_{kl}$ with respect to r is 0, Equation (5) is reduced to

(6) $${𝛻}_{j}C_{ijkl}^{0}{\delta \varepsilon }_{kl}\left(r\right)=-{𝛻}_{j}\delta C_{ijkl}\left(r\right){\bar{\varepsilon }}_{kl}.$$

In order to solve the heterogeneous strain field ${\delta \varepsilon }_{kl}\left(r\right)$, one should first go back to the displacement field by using

(7) $$\varepsilon_{ij}\left(r\right)=\frac{1}{2}\left(\frac{\partial u_{i}\left(r\right)}{\partial r_{j}}+\frac{\partial u_{j}\left(r\right)}{\partial r_{i}}\right),$$

where $u_{i}\left(r\right)$ denotes the *i*th component of the displacement field.

The displacement field $u_{x}\left(r\right)$ and $u_{y}\left(r\right)$ can be written as

(8) $$\left\{\begin{array}{c} u_{x}\left(r\right)={\bar{\varepsilon }}_{xx}\cdot r_{x}+{\bar{\varepsilon }}_{xy}\cdot r_{y}+\delta u_{x}\left(r\right)\\ u_{y}\left(r\right)={\bar{\varepsilon }}_{xy}\cdot r_{x}+{\bar{\varepsilon }}_{yy}\cdot r_{y}+\delta u_{y}\left(r\right) \end{array},\right.$$

where $\delta u_{x}\left(r\right)$ and $\delta u_{y}\left(r\right)$ are the deviations of the displacement fields from the one induced by the homogeneous strain $\bar{\varepsilon }$.

By using Equations (7) and (8), the first-order approximation in Equation (6) is rewritten using the δu fields as

(9) $$C_{ijkl}^{0}\frac{\partial^{2}{\delta u}_{k}\left(r\right)}{\partial r_{j}\partial r_{l}}=-{\bar{\varepsilon }}_{kl}\frac{\partial \delta C_{ijkl}\left(r\right)}{\partial r_{j}},$$

The first-order $\delta u\left(r\right)$ fields are obtained by solving Equation (9) in the Fourier space:

(10) $${\tilde{\delta u}}_{k}^{1}\left(k\right)=-IG_{ik}{\bar{\varepsilon }}_{kl}k_{j}{\tilde{\delta C}}_{ijkl}\left(k\right),$$

where ${\tilde{\delta u}}_{k}^{1}\left(k\right)$ and ${\tilde{\delta C}}_{ijkl}\left(k\right)$ are the Fourier transformations of the first order approximation ${\delta u}_{k}\left(r\right)$ and $\delta C_{ijkl}\left(r\right)$, respectively, $k_{j}$ is the $j^{th}$ component of the k vector in the Fourier space, $G_{ij}$ is the Green’s tensor whose inverse is defined as $G_{ik}^{-1}=C_{ijkl}^{0}k_{j}k_{l}$, and I is the imaginary unit.

The first-order heterogeneous strain field $\delta \varepsilon_{kl}^{1}\left(r\right)$ is then obtained using Equations (2), (7) and (8). To obtain the strain field beyond the first-order approximation. One needs to rewrite Equation (5) as

(11) $${𝛻}_{j}C_{ijkl}^{0}{\delta \varepsilon }_{kl}^{n+1}\left(r\right)=-{𝛻}_{j}\delta C_{ijkl}\left(r\right){\bar{\varepsilon }}_{kl}-{𝛻}_{j}\delta C_{ijkl}\left(r\right)\delta \varepsilon_{kl}^{n}\left(r\right),$$

where the superscript of the heterogeneous strain indicates the order of the approximation. Equation (11) can also be written using $\delta u\left(r\right)$ as

(12) $$C_{ijkl}^{0}\frac{\partial^{2}{\delta u}_{k}^{n+1}}{\partial r_{j}\partial r_{l}}=-\frac{\partial }{\partial r_{j}}\left[\delta C_{ijkl}\left(r\right){\bar{\varepsilon }}_{kl}+\delta C_{ijkl}\left(r\right)\delta \varepsilon_{kl}^{n}\left(r\right)\right].$$

High-order approximations for the heterogeneous strain field ${\delta \varepsilon }_{kl}\left(r\right)$ can be obtained from $\delta \varepsilon_{kl}^{n}\left(r\right)$ using Equation (12). The solution to ${\left(n+1\right)}^{th}$ $\delta u_{k}\left(r\right)$ in the Fourier space is then

(13) $${\tilde{\delta u}}_{k}^{n+1}\left(k\right)=-IG_{ik}k_{j}{\tilde{h}}_{ij}\left(k\right),$$

where ${\tilde{h}}_{ij}\left(k\right)$ is the Fourier transform of $\delta C_{ijkl}\left(r\right){\bar{\varepsilon }}_{kl}+\delta C_{ijkl}\left(r\right)\delta \varepsilon_{kl}^{n}\left(r\right)$. It is shown in ref. [22] that a reasonably accurate solution of the strain field can be obtained within 10 iterations of Equation (13). While the local strains obtained using this method only considered elastic deformation, it is a reasonable approximation to strongly strengthened nanocrystalline materials.

### 2.2. Phase-Field Model of Deformation Twinning

A twin structure consists of two regions with identical crystallographic orientation, but with a mirror-image stacking sequence. The two mirror crystal structures are separated by a planar defect known as the twin boundary. Deformation twinning is the formation of twin boundaries from the cooperative shift of lattice atoms under external shear stress [23,24]. Upon the application of external shear stress to a material, partial dislocations can arise, which represents lattice defects gliding along specific crystallographic planes. The overlap and accumulation of partial dislocations can lead to the formation of a stacking fault. If the stacking fault energy is sufficiently low, this stacking fault can propagate throughout the lattice and result in the formation of a twin. Typically, deformation twinning occurs in some face-centered cubic (FCC) metals with low stacking fault energy, such as silver and copper. Many previous works were dedicated to understanding the nucleation and growth processes of deformation twins, including a particular dislocation arrangement that induced twinning nucleation [25,26,27,28,29], theoretical study on the strain due to twinning [30], and density functional theory calculations of twinning energy pathways [31]. It has been used extensively in grain-boundary engineering to improve the mechanical properties of polycrystalline materials under the influence of metallurgical variables, such as temperature [32,33,34], strain rate [24,35], grain size and texture and crystallographic order [24].

Materials with higher stacking fault energy can also activate twinning when the grain size decreases to nanoscale. In nanocrystalline materials, deformation twinning can occur through both heterogeneous and homogeneous mechanisms [36,37]. The heterogeneous mechanism involves the emission of partial dislocations from the grain boundaries onto neighboring slip planes, while the homogeneous mechanism takes place in the grain interiors by a nucleation mechanism involving the dynamical overlap of the stacking faults of dislocations.

While several mechanisms may lead to the nucleation of the twin boundaries, two essential steps, nucleation of new twinning partial dislocations and gliding of existing twinning partial dislocations, are related to a further growth of the twins. Normal to the existing twin boundary, nucleation of new twinning partials is needed to thicken the twins. Along the existing twin boundary, the twinning partial dislocations need a large enough shear stress to glide through the lattice and further grow the twin boundary to a larger area. PF models [7,38,39] have been developed to study the growth of deformation twins and the evolution of the twinning structures. As a coarse-grain model, the PF method did not explicitly resolve the atomic structure of twins; instead, it incorporated the surface energy for the twin boundary and the barrier for nucleating new twin layers (through nucleation of partial dislocations) from atomistic calculations [40] and predicted twin structures comparable to experiment observations [39]. It is assumed in the PF model that the nucleation of partial dislocations is due to the instability of the crystal structure under strong local shear stress.

**Figure 2:**
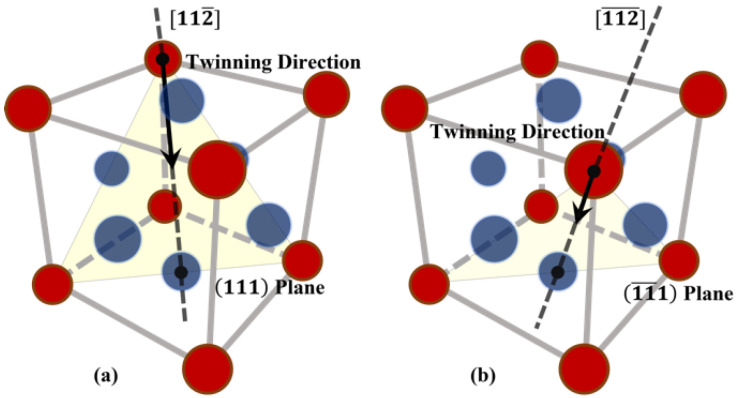
The crystallographic description of the twinning process in (**a**) $\left(111\right)$ habit plane along $\left[11\bar{2}\right]$ direction, (**b**) $\left(\bar{11}1\right)$ habit plane along $\left[\bar{112}\right]$ direction. The red circles represent the atoms in the corners of the FCC lattice while the dark-blue circles represent the face-centered atoms. The two twinning directions are indicated by black dashed lines.

**Figure 3:**
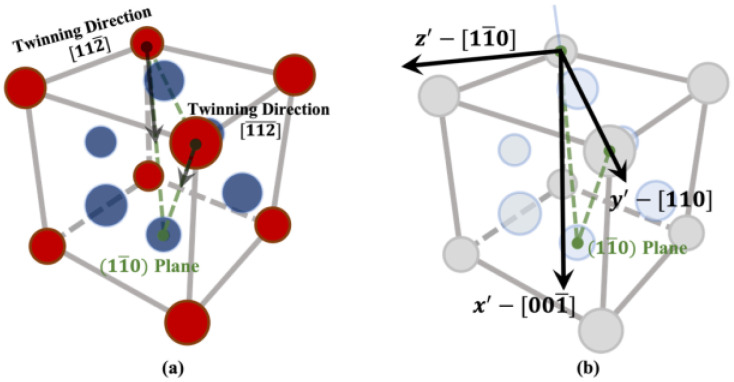
(**a**) Two twinning directions $\left[11\bar{2}\right]$ & $\left[\bar{112}\right]$ and their common plane $\left(1\bar{1}0\right)$, (**b**) The simulation plane. It is the $x^{\prime }-y^{\prime }$ plane contains the common plane $\left(1\bar{1}0\right)$. The same coloring scheme in Figure 2 is applied in (**a**), while the atom colors were tuned down in (**b**) in order to provide a clear view of the simulation plane.

**Figure 4:**
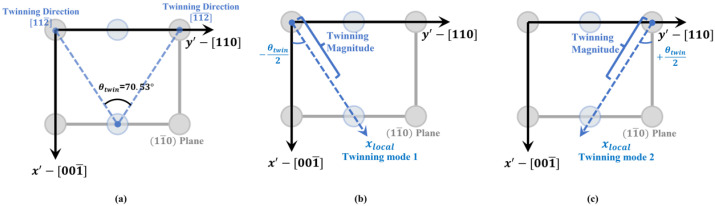
2D view of (**a**) The simulation frame and the two twinning directions; (**b**) the local reference frame for twinning mode $\left(111\right)[11\bar{2}]$. The angle between $x_{ref}$ and $x^{\prime }$ is $-\theta_{twin}/2$; (**c**) the local reference frame for twinning mode $\left(\bar{11}1)[\bar{112}\right]$. The angle between $x_{ref}$ and $x^{\prime }$ is $+\theta_{twin}/2$. The gray circles represent the atoms in the corners of the FCC lattice while the light-blue circles represent the face-centered atoms. The angle between the two twinning directions is $\theta_{twin}=70.53{}^{\circ}$. The twinning magnitude is $\frac{1}{\sqrt{2}}$. for the FCC structure.

There are 12 possible twinning modes in FCC metals which may take place on $\left\{111\right\}$ habit planes along $\langle 112\rangle$ directions. In Figure 2, the crystallographic descriptions of the two twinning modes $\left(111)[11\bar{2}\right]$ and $\left(\bar{11}1)[\bar{112}\right]$ are shown. Since both the twinning modes are in the plane $\left(1\bar{1}0\right)$, for the purpose of identifying the critical strain for twin growth, it is enough to only focus on these two modes and carry out a two-dimensional (2D) calculation on this plane, instead of performing a costly 3-dimensional calculation resolving all the 12 modes. The two modes considered in the simulations are shown in Figure 3a, and the 2D simulation domain is the $x^{\prime }-y^{\prime }$ plane shown in Figure 3b. A 2D view of the simulation domain and the two twinning directions on the $\left(1\bar{1}0\right)$ plane is shown in Figure 4.

Within the 2D simulation plane, the crystal may stay in the twinning state or the undeformed state. A spatially dependent order parameter field $\eta_{1}(r)$, which takes 0 in the undeformed state and 1 in the twinning state, is introduced to characterize the twinning region for the $\left(\bar{11}1)[\bar{112}\right]$ twinning mode. Similarly, $\eta_{2}(r)$ is introduced for the $\left(111\right)[11\bar{2}]$ twinning mode. The local twinning strains $\gamma_{\left(111\right)[11\bar{2}]}(r)$ and $\gamma_{\left(\bar{11}1)[\bar{112}\right]}(r)$ can then be expressed using $\eta_{1}\left(r\right)$ and $\eta_{2}\left(r\right)$ by the following relations:

(14) $$\begin{array}{c} \gamma_{\left(111\right)[11\bar{2}]}\left(r\right)=\eta_{1}\left(r\right)\cdot \gamma_{\left(111\right)[11\bar{2}]}^{twin}\\ \gamma_{\left(\bar{11}1)[\bar{112}\right]}\left(r\right)=\eta_{2}\left(r\right)\cdot \gamma_{\left(\bar{11}1)[\bar{112}\right]}^{twin} \end{array}$$

where $\gamma_{plane\cdot direction}^{twin}$ is the shear strain of the fully twinned state in the corresponding twinning mode.

By defining a reference frame where the x-axis is along the twinning direction (the $x_{local}$ shown in Figure 4b,c and the y-axis is along the normal direction to the habit plane), the twinning strain of the two modes can be expressed using pure shear strain tensors as follows:

$$\varepsilon_{ij,ref}^{twin,1}=\left[\begin{array}{cc} 0 & \frac{\gamma_{twin}}{2}\\ \frac{\gamma_{twin}}{2} & 0 \end{array}\right]\;\mathrm{and}\;\varepsilon_{ij,ref}^{twin,2}=\left[\begin{array}{cc} 0 & \frac{-\gamma_{twin}}{2}\\ \frac{{-\gamma }_{twin}}{2} & 0 \end{array}\right],$$

where $\gamma_{twin}$ is the magnitudes of the shear strain which is $1/\sqrt{2}$ for the FCC structure [30]. The strain $\varepsilon_{plane\cdot direction}^{twin}$ in the simulation frame $x^{\prime }-y^{\prime }$ is related to the reference frame shear strain by a rotation of $-\theta_{twin}/2$ for the twinning mode $\left(111\right)[11\bar{2}]$ and a rotation of $\theta_{twin}/2$ for the twinning mode $\left(\bar{11}1)[\bar{112}\right]$. The strain tensors $\varepsilon_{ij}^{twin}$ in the simulation frame are obtained by rotating the pure shear strain tensors in the reference frame around the z axis with $-\theta_{twin}/2$ and $\theta_{twin}/2$, correspondingly:

(15) $$\left\{\begin{array}{c} \varepsilon_{ij}^{twin,1}=\left[\begin{array}{cc} 2\cos\left(-\frac{\theta }{2}\right)\sin\left(-\frac{\theta }{2}\right)\frac{\gamma_{twin}}{2} & \left[\cos^{2}\left(-\frac{\theta }{2}\right)-\sin^{2}\left(-\frac{\theta }{2}\right)\right]\frac{\gamma_{twin}}{2}\\ \left[\cos^{2}\left(-\frac{\theta }{2}\right)-\sin^{2}\left(-\frac{\theta }{2}\right)\right]\frac{\gamma_{twin}}{2} & -2\sin\left(-\frac{\theta }{2}\right)\cos\left(-\frac{\theta }{2}\right)\frac{\gamma_{twin}}{2} \end{array}\right]\\ \varepsilon_{ij}^{twin,2}=\left[\begin{array}{cc} -2\cos\left(\frac{\theta }{2}\right)\sin\left(\frac{\theta }{2}\right)\frac{\gamma_{twin}}{2} & -\left[\cos^{2}\left(\frac{\theta }{2}\right)-\sin^{2}\left(\frac{\theta }{2}\right)\right]\frac{\gamma_{twin}}{2}\\ -\left[\cos^{2}\left(\frac{\theta }{2}\right)-\sin^{2}\left(\frac{\theta }{2}\right)\right]\frac{\gamma_{twin}}{2} & 2\sin\left(\frac{\theta }{2}\right)\cos\left(\frac{\theta }{2}\right)\frac{\gamma_{twin}}{2} \end{array}\right] \end{array}\right.$$

To track the growth of the twinning region, a PF free energy functional considering the two twinning modes and the corresponding elastic energy is given by

(16) $$F=\int_{\Omega }^{}\left[f\left(\eta_{1},\eta_{2}\right)+\sum_{p=1,2}\frac{\kappa_{p,ij}}{2}{𝛻}_{i}\eta_{p}{𝛻}_{j}\eta_{p}+E_{ela}\right]dV,$$

where f describes the energy landscape along the twinning direction, $\frac{\kappa_{p,ij}}{2}\nabla_{i}\eta_{p}\nabla_{j}\eta_{p}$ is the interfacial energy between the twinned structure and the original crystal for the $p^{th}$ twinning mode, $E_{ela}$ is the elastic energy induced by twinning, Ω represents the domain of interest. In the following sections, the meanings of these terms are discussed in detail.

#### 2.2.1. Energy Landscape

The energy landscape term f in Equation (16) incorporates the energy barrier between the original undeformed state and the twinning state. It has been shown in the work of Kibey et al. [40], the energy related to forming a n+1 layer twin structure has two parts, the surface energy of the 2 twin boundaries $2\gamma_{tsf}$, and the barrier $\gamma_{ut}$ for nucleating the n+1 layer on the n-layer twin. For a twin structure with n≥3, the twin nucleation barrier $\gamma_{ut}$ becomes a constant, and the energy landscape along the twinning direction forms a symmetric double well. For a single-mode twinning system, the double-well energy landscape can be approximated by the following polynomial form

(17) $$f\left(\eta \right)=∆f_{max}[A_{0}+A_{2}{\left(\eta -0.5\right)}^{2}+A_{4}{\left(\eta -0.5\right)}^{4}+A_{6}{\left(\eta -0.5\right)}^{6}\;+A_{8}{\left(\eta -0.5\right)}^{8}]$$

where the factor $∆f_{max}$ is the energy difference between the twin nucleation barrier $\gamma_{ut}$ and the surface energy $2\gamma_{tsf}$, the fitting parameters in the polynomial ($A_{0}$, $A_{2}$, $A_{4}$, $A_{6}$ and $A_{8}$) are chosen such that the energy at the twinning state (η=1) and the undeformed state (η=0) are zero and the barrier height between the two states is $∆f_{max}$. For a two-mode twinning system, the double-well energy landscape can be constructed as follows

(18) $$\begin{array}{cc} \left(\eta_{1},\eta_{2}\right)=\Delta f_{\max} & \left[A_{0}+A_{2}\sum_{p=1,2}{\left(\eta_{p}-0.5\right)}^{2}+A_{4}\sum_{p=1,2}{\left(\eta_{p}-0.5\right)}^{4}\right.\\ \; & \left.+A_{6}\sum_{p=1,2}{\left(\eta_{p}-0.5\right)}^{6}+A_{8}\sum_{p=1,2}{\left(\eta_{p}-0.5\right)}^{8}\right]\\ \; & +A_{\gamma }\sum_{p,q(p\neq q)}\eta_{p}^{2}\eta_{q}^{2}, \end{array}$$

where a new term $A_{\gamma }\sum_{p,q\left(p\neq q\right)}\eta_{p}^{2}\eta_{q}^{2}$ is added to describe the interaction between different twinning modes.

#### 2.2.2. Interfacial Energy

The interface between the twinning region and the undeformed matrix is defined by a transition region where $\eta_{p}$ varies from 0 to 1. The term $\sum_{p=1,2}\frac{\kappa_{p,ij}}{2}{𝛻}_{i}\eta_{p}{𝛻}_{j}\eta_{p}$ in Equation (16) is used to account for the sum of the surface energies for two twinning modes. For a commonly seen prime-shape twin structure, its interface can be separated into two parts: the twin boundaries parallel to the growth direction and the dislocations at the end of the twin [39]. The dislocation cores can be approximated as incoherent interfaces which have much larger surface energy compared to the twin boundaries [36]. This strong surface energy anisotropy can be incorporated using a gradient energy coefficient tensor in the reference frame

$$\kappa_{p,ij}^{ref}=\left[\begin{array}{cc} \kappa_{11} & 0\\ 0 & \kappa_{22} \end{array}\right],$$

where $\kappa_{11}$ and $\kappa_{22}$ incorporates the surface energies along the twinning direction and the normal direction of the twinning. The value of $\kappa_{11}$ and $\kappa_{22}$ can be determined by the dislocation core energy and twin boundary energy, respectively, which can be obtained from atomic simulations and DFT calculation [39].

To calculate the term $\frac{\kappa_{p,ij}}{2}{𝛻}_{i}\eta_{p}{𝛻}_{j}\eta_{p}$ in the simulation frame, the reference frame gradient energy coefficient tensor must be rotated to the simulation frame. The gradient energy coefficient tensor $\kappa_{p,ij}^{}$ in the simulation frame $x^{\prime }-y^{\prime }$ is related to $\kappa_{p,ij}^{ref}$ in the reference frame by a rotation of $-\theta_{twin}/2$ for the twinning mode $\left(111\right)[11\bar{2}]$ and a rotation of $\theta_{twin}/2$ for the twinning mode $\left(\bar{11}1)[\bar{112}\right]$. The rotation matrix is similar to the one used for rotating the strain tensor in Equation (15).

#### 2.2.3. Elastic Energy

The elastic energy term in Equation (16) is

(19) $$E_{ela}=\frac{1}{2}C_{ijkl}^{\prime }\left(\varepsilon_{ij}-\varepsilon_{ij}^{0}\right)\left(\varepsilon_{kl}^{}-\varepsilon_{kl}^{0}\right),$$

where $C_{ijkl}^{\prime }$ is the elastic moduli tensor in the simulation frame, $\varepsilon_{ij}$ is the total strain tensor, $\varepsilon_{ij}^{0}$ is the eigenstrain tensor related to the twinning strain. The eigenstrain is defined as

(20) $$\varepsilon =\sum_{p}\varepsilon_{ij,ref}^{twin,p}H\left(\eta_{p}\right),$$

This is a summation of the twinning-induced strains from the two twinning modes with a weight function $H(\eta_{p})$ given as

(21) $$H\left(\eta_{p}\right)=-2\eta_{p}^{3}+3\eta_{p}^{2},$$

This weight function ensures that the derivative of the twinning-induced strain becomes zero in the twinning and the undeformed states in the dynamic equations presented later.

The total strain $\varepsilon_{ij}$ can be separated into two parts as Equation (2). ${\bar{\varepsilon }}_{ij}$ is the homogeneous strain representing the macroscopic deformation of the grain, and the heterogeneous strain ${\delta \varepsilon }_{ij}$ refers to the local strain deviation. The heterogeneous strain should follow $\int \delta \varepsilon_{ij}dV=0$.

To obtain the strain field during the twinning process, the mechanical equilibrium equation

(22) $${𝛻}_{j}\sigma_{ij}={𝛻}_{j}C_{ijkl}^{\prime }\left({\bar{\varepsilon }}_{kl}^{}+\delta \varepsilon_{kl}-\varepsilon_{kl}^{0}\right)=0,$$

is solved using the Fourier spectral method [41].

Using Equations (7), (8) and (19), Equation (22) becomes

(23) $$C_{ijkl}^{\prime }k_{j}k_{l}{\tilde{\delta u}}_{k}\left(k\right)=C_{ijkl}^{\prime }k_{j}\varepsilon_{kl}^{0}\left(r\right)$$

in the Fourier space, where ${\tilde{\delta u}}_{k}\left(k\right)$ is the Fourier transformations of the heterogeneous displacement ${\delta u}_{k}\left(r\right)$ and $k_{j}$ is the *j*th component of the k vector. The strain field can then be derived based on Equation (7).

#### 2.2.4. Order Parameters Evolution

The evolution of the twinning order parameters is calculated based on the time-dependent Ginzburg–Landau (TDGL) equation

(24) $$\frac{\partial \eta_{p}}{\partial t}=-L\left(\frac{\partial f\left(\eta_{p}\right)}{\partial \eta_{p}}-\kappa_{p,ij}{𝛻}_{i}{𝛻}_{j}\eta_{p}+\frac{\partial E_{ela}}{\partial \eta_{p}}\right)$$

where L is a kinetics coefficient, t is time.

Since the total deformation of the system is an input parameter which is a constant during the evolution of the twin structure, a penalty term

(25) $$P=\sum_{ij}M_{ij}\left[\left(\frac{1}{V}\int \varepsilon_{ij}^{0}dV-{\bar{\varepsilon }}_{ij}\right)\left(\varepsilon_{ij}^{twin,p}\frac{1}{V}\int \frac{\partial H\left(\eta_{p}\right)}{\partial \eta_{p}}dV\right)\right],$$

where $M_{ij}$ are a penalty constant, is added to the free energy term. Equation (24) becomes

(26) $$\frac{\partial \eta_{p}}{\partial t}=-L\left(\frac{\partial f\left(\eta_{p}\right)}{\partial \eta_{p}}-\kappa_{p,ij}{𝛻}_{i}{𝛻}_{j}\eta_{p}+\frac{\partial E_{ela}}{\partial \eta_{p}}-P\right).$$

In the Fourier space, Equation (26) becomes

(27) $$\frac{\partial {\tilde{\eta }}_{p}(k,t)}{\partial t}=-L\left[{\left(\frac{\partial \tilde{f}\left(\eta_{p}\right)}{\partial \eta_{p}}\right)}_{k}+\kappa_{p,ij}k_{i}k_{j}{\tilde{\eta }}_{p}\left(k,t\right)+{\left(\frac{\partial {\tilde{E}}_{ela}}{\partial \eta_{p}}\right)}_{k}\right]-L{\tilde{P}}_{k}$$

where $k=\left(k_{1},k_{2}\right)$ is a vector in the Fourier space, ${\tilde{\eta }}_{p}(k,t)$*,* ${\left[\frac{\partial \tilde{f}\left(\eta_{p}\right)}{\partial \eta_{p}}\right]}_{k}$*,* ${\left(\frac{\partial {\tilde{E}}_{ela}}{\partial \eta_{p}}\right)}_{k},$ and $\tilde{P_{k}}$ represent the Fourier transform of the twin order parameter, the derivative of the local energy density function, the derivative of local elastic energy, and the penalty term, respectively.

Equation (27) is then approximated using the following semi-implicit Fourier spectral method

(28) $${\tilde{\eta }}_{p,k}^{n+1}=\frac{{\tilde{\eta }}_{p,k}^{n}-L∆t\left[{{\left(\frac{\partial \tilde{f}\left(\eta_{p}\right)}{\partial \eta_{p}}\right)}^{n}}_{k}-{{\left(\frac{\partial {\tilde{E}}_{ela}}{\partial \eta_{p}}\right)}^{n}}_{k}-{\tilde{P}}_{k}^{n}\right]}{1+L∆t\kappa_{p,ij}k},$$

where ∆t is the time step size, and the superscripts n and n+1 indicate the field at time step n and n+1.

## 3. Results and Discussion

In this work, the growth of the deformation twins in three FCC metals, aluminum, nickel, and copper, aisre examined. Although aluminum has a relatively high stacking fault energy, deformation twinning in nanocrystalline aluminum has also been well-recognized [36]. The elastic constants C, twin nucleation barriers $\gamma_{ut}$ and the stacking fault energy $\gamma_{tsf}$ for the three materials [40] are listed in Table 1.

The evolution of the twinning structure is tracked by solving Equation (28) under the equilibrated strain field through Equation (23). All the calculations were conducted in a square domain with 1024∆x×1024∆y grids where the grid size ∆x=∆y=0.1 nm with periodic boundary conditions. A PF model length scale $l=\frac{1}{10}\sqrt{\frac{\kappa_{11}}{\left|∆f_{max}\right|}}=0.2$ nm is used to characterize the thickness of the transition layer of η at the twin-matrix boundary where $∆f_{max}$ is given in Equation (17). The energy landscape function f in the energy functional Equation (16) was non-dimensionalized as $f^{*}=\frac{f}{\left|∆f_{max}\right|}$, and the values of coefficients were set to $A_{0}=1.0$, $A_{2}=-12.43$, $A_{4}=61.17$, $A_{6}=-152.31$, $A_{8}=166.45$ for all three materials (note: $∆f_{max}$ is different for the 3 materials). For the deformation twinning simulation in aluminum, its dimensionless gradient coefficients were $\kappa_{11}^{*}=\frac{\kappa_{11}}{l^{2}\left|∆f_{max}\right|}=112$ and $\kappa_{22}^{*}=\frac{\kappa_{22}}{l^{2}\left|∆f_{max}\right|}=0.09$, its elastic constants were adopted from $C_{11}^{*}=\frac{C_{11}}{\left|f_{max}\right|}=114$*,* $C_{12}^{*}=\frac{C_{12}}{\left|f_{max}\right|}=62$ *and* $C_{44}^{*}=\frac{C_{44}}{\left|∆f_{max}\right|}=32$. The dimensionless parameters of the other two materials are calculated in the same approach and listed in Table 2. The dimensionless numerical parameters for solving Equation (28) were $∆x^{*}=\frac{∆x}{l}=0.5$, $∆t^{*}=L\left|∆f_{max}\right|∆t=10^{-4}$. For the penalty constants in Equation (25), $M_{11}=1000$, $M_{12}=M_{21}=4000$ and $M_{22}=1000$ were used.

**Figure 5:**
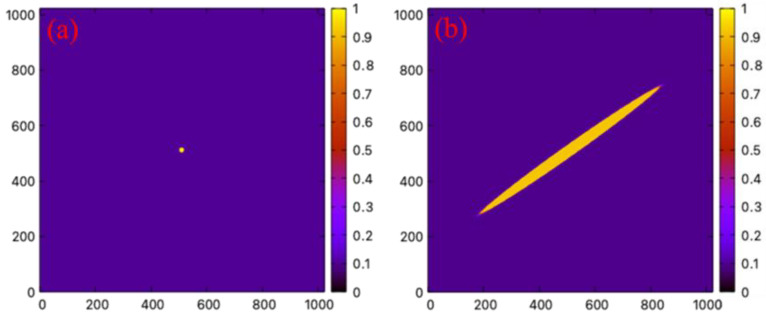
The twinning order parameter profile of a single twin growth under $0.1\cdot \varepsilon_{ij}^{twin,1}$ at (**a**) the initial state (**b**) $50,000∆t^{*}$. As shown in the color bar, the yellow part corresponds to the twinning region with the order parameter η = 1, the purple part corresponds to the original lattice with the order parameter η = 0.

To validate our numerical model, the theoretically predicted twinning direction was first reproduced. An initial twin may grow or shrink depending on the magnitude of the local shear strain in the reference frame. For a single crystal, the local shear strain can be controlled by applying a homogenous strain $\alpha \cdot \varepsilon_{ij}^{twin,1}$, with the factor α controlling the magnitude, to the simulation box which can be transformed to pure shear in the reference frame by rotating $\theta_{twin}/2$. To verify the model, the growth of a single twin in Al under a fixed homogeneous strain with the magnitude factor α=0.1 was carried out. The growth of the twin structure is shown in Figure 5. A circular-shaped twin domain of radius 5∆x was initialized at the center of the system (as shown in Figure 5a). A bi-convex lenticular twinning shape commonly seen in experiments is obtained after 50,000 time steps (as shown in Figure 5b). The angle between the twinning growth direction and the horizontal direction is 35.4° which is consistent with the crystallographic theory illustrated in Figure 4.

A critical strain magnitude can be obtained by applying $\alpha \cdot \varepsilon_{ij}^{twin,1}$ to the simulation box with an existing lenticular twin structure and identify the threshold α below which the twin structure will disappear. The critical shear strains and the corresponding magnitude parameter α for Al, Ni, and Cu are obtained through multiple simulations and are listed in Table 3.

In a polycrystalline system, the deformation twinning may happen in the region where the shear strain in the grain interiors surpasses the critical shear strain shown in Table 3. Previous research on nanocrystalline systems has revealed that as the grain size approaches approximately 10 nm [42,43,44,45], the dominant deformation mode transitions from dislocation slip, commonly observed in coarse-grained metals, to twinning. Thus, it is possible to establish a correlation between the local shrinkage-induced deformation and potential regions of twinning.

The method introduced in the thermal strain inhomogeneity section is used here to estimate the shear strain in the polycrystals induced by thermal shrinkage. There is typically a large temperature drop involved in rapid solidification processes, the thermal expansion coefficients become temperature-dependent in this case [40,41,46]. The total strains from the thermal shrinkage over the large temperature range can be estimated by integrating the expansion coefficient over that relevant temperature range. For Al, Ni, and Cu, the total hydrostatic strain from the thermal shrinkage, when cooling the materials from the melting point to the room temperature, are $\varepsilon_{Al}^{0}=-0.0187$, $\varepsilon_{Ni}^{0}=-0.0232$ and $\varepsilon_{Cu}^{0}=-0.0203$. These strains are then applied to the polycrystalline structure shown in Figure 1.

**Figure 6:**
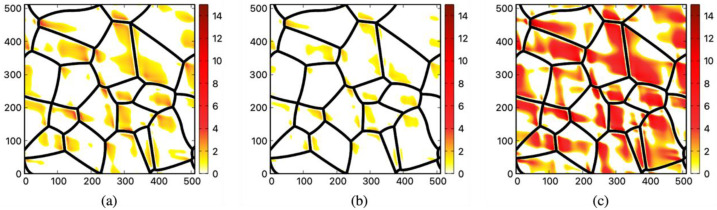
Potential deformation twinning regions in the nanocrystalline structure for (**a**) Al, (**b**) Cu, and (**c**) Ni when the value of GB shear modulus is set at 30% of the bulk value. The original grain boundaries are shown in black; the highlighted regions are the potential deformation twinning regions where the local shear strain is larger than the critical shear strain for the growth of deformation twins. The color bar corresponds to the multiple of the local shear strain relative to the critical shear strain. Only the regions with the local shear strain larger than the critical shear strain are colored.

**Figure 7:**
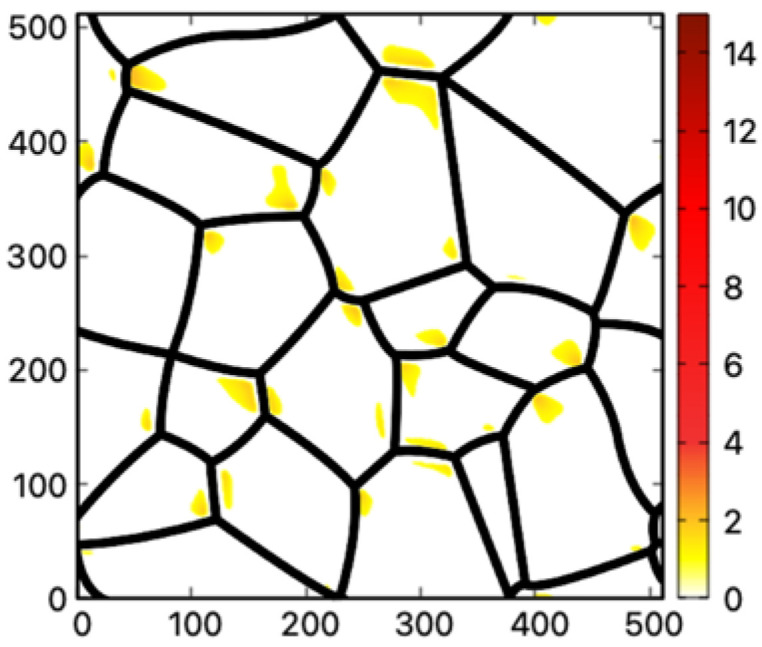
Potential deformation twinning regions in the nanocrystalline structure for Ni when the value of GB shear modulus is set at 50% of the bulk value. The color scheme in Figure 6 is used.

Given the significant distinction between GBs and bulk, coupled with the sensitivity of GBs to temperature changes, it becomes imperative to assign different modulus values when simulating a homogeneous strain on the polycrystalline system. Considering the presence of various mechanisms such as pre-melting and segregation, which can significantly influence the elastic behavior of GBs, certain approximations must be made regarding the GB elastic properties. In this particular study, an approximation approach was employed, where the shear modulus in the GB region was set as a fixed fraction of the shear modulus in the grain interiors. Three sets of simulations were performed, each with varying values for the grain boundary (GB) shear modulus. In these simulations, the GB shear modulus was assigned as 30%, 50%, and 70% of the bulk value, respectively. Additionally, the GB bulk modulus was uniformly set to 50% of the bulk value. By extracting shear strains from the simulations within the grain interiors, a comparison was made with the critical shear strains specified in Table 3 for the three materials. Regions in which the shear strain in the grain interiors exceeded the critical shear strain for twinning were visualized in Figure 6 and Figure 7.

When the value of shear modulus is set at 30% of the bulk value, all three materials report possible twinning regions near the junction of the GBs, as shown in Figure 6. Among the materials examined, nickel has the largest fraction of possible twinning regions. The local strain induced by thermal shrinkage can be remarkably significant in certain areas, reaching up to 14 times the critical shear strain. Among the materials studied, Nickel stands out as the only material that exhibits potential twinning regions when the shear modulus of grain boundaries (GBs) is set at 50% of the bulk value. The distinctive behavior exhibited by Nickel can be ascribed to its high melting point of 1445 ℃ and its relatively low critical twinning shear strain. Nickel’s superior melting temperature compared to copper and aluminum enables it to undergo a more pronounced thermal shrinkage ($\varepsilon_{Ni}^{0}=-0.0232$) subsequent to quenching. Furthermore, Nickel possesses a relatively reduced critical shear strain ($\varepsilon_{Ni}^{twinning}=0.0042$) conducive to deformation twinning. These properties enable Nickel to undergo a more pronounced response to thermal shrinkage, thereby facilitating more occurrences of twinning regions. It is noted that the activation of other deformation mechanisms may significantly reduce the magnitude of the thermal- hrinkage-induced shear in the polycrystalline structure; experimental evidence [47] indicates that the large critical shear strain from Table 3 can be reached at least in Nickel alloys.

In copper, although twinning regions are present, their fraction is considerably smaller compared to Nickel. The simulative potential twinning in Nickel and Copper is supported by Wang et al. [5], who observed dense dislocations and twinning in the substructures of Ni-Cu alloys under high undercooling conditions. In the case of aluminum, one can also identify large regions where the thermal shrinkage-induced local strain exceeded the critical shear. This is only possible for nanocrystalline aluminum where deformation through dislocation motions is strongly suppressed. In regular polycrystalline aluminum, the critical shear for deformation twinning cannot be reached since the dislocation cross slip may happen at a much smaller shear strain [36,44,45,47,48,49,50,51].

When the value of the shear modulus of GBs is set at 70% of the bulk value, all three materials show no potential twinning region, which is much different compared to a 30% case. The explanation for this phenomenon can be attributed to multiple factors. Firstly, grain boundaries with higher shear modulus exhibit greater resistance to deformation, making them less susceptible to the thermal shrinkage-induced strain which is the primary driving force for twinning. Secondly, the shear modulus of grain boundaries influences the transmission of shear stress across them. Higher shear modulus at grain boundaries, closer in value to that of the grain interior, facilitates a more efficient load transfer and redistribution between neighboring grains, thereby promoting a more homogeneous distribution of strain. Consequently, in the case where the grain boundary shear modulus is set to 70% of the bulk value, the resulting local strain is more uniform and of a smaller magnitude. This uniform and limited strain distribution ultimately precludes the emergence of potential deformation twinning in all three materials. Since the deformation twinning in the results depends strongly on the grain boundary shear modulus, one might be able to validate the results by observing the twinning behavior of materials with a trace amount of alloying element that segregates strongly to the GB area, therefore modifying the GB shear properties.

The simulative findings emphasize the significance of deformation twinning, which predominantly occurs near grain boundaries. This phenomenon is particularly pronounced in materials such as nickel, with high melting points and relatively low critical shear strains. Understanding the prevalence and behavior of deformation twinning provides insights for real-world applications. Twin boundaries act as effective barriers to dislocation motion, resulting in improved strength, hardness, and ductility of the material. By carefully selecting the appropriate material or alloy, it is possible to anticipate a higher prevalence of twinning boundaries within the material. Materials can be designed to exhibit enhanced strength, ductility, and strain-hardening capabilities. For instance, Zhang et al. [52] conducted a study on stainless steel films produced via Magnetron-sputter deposition. They found that the films, characterized by a high density of twins along the {111} crystallographic plane, exhibited a hardness approximately one order of magnitude higher than that of bulk stainless-steel films. This indicates that the introduction of twin boundaries can significantly enhance the mechanical properties of the material, leading to a substantial increase in hardness. Furthermore, it opens up possibilities for intentionally inducing physical shrinkage to promote deformation twinning as a strategy for enhancing material properties. These research outcomes are instrumental in guiding the design and optimization of materials, ultimately leading to improved performance and reliability in practical applications.

It is noted that the current model did not explicitly consider the effect of the semi-solid mushy zone which has been extensively studied in previous works [53,54,55]. Shrinkage-induced stress within intergranular liquid channels in the mushy zone has long been related to the formation of solidification defects [56,57]. Due to the limitation of the method used in this work, this important contribution of stress within the microstructure was not considered. However, it has been shown that considering the mushy zone will significantly increase the stress inhomogeneity in the microstructure which might be added on top of the macroscopic stress distribution in the materials [53]. One should also note that the grain size in this work is controlled by the interface thickness in the PF model used in the calculation of thermal shrinkage induce strain. It has been shown that this interface thickness can be chosen to be much larger than the physical thickness of grain boundaries as long as the interface region in the model is much smaller than the grain interior in the model [58]. From this point of view, the grain size used here could be any size larger than a couple of nm which is set by the ratio of the grid points between the grain interior and the GB in the model. However, since the model did not consider the role of dislocations in the deformation process, the strain calculation can only be close to realistic for grain sizes smaller than 100 nm.

## 4. Conclusions

The deformation twinning mechanism for the formation of twin structures in rapid solidification processes is examined numerically in this work. Critical shear strains for the growth of deformation twins were calculated for Ni, Cu, and Al using a PF approach. The critical strain is then compared with the thermal shrinkage-induced strain in polycrystalline structures. The main findings are summarized below.

1. For Ni, the calculated critical shear strain for deformation twinning is about 0.4% which is similar to the experimentally observed residual strain.
2. The formation of deformation twins is strongly affected by the shear modulus of the GBs.
3. When the GB shear resistance is significantly reduced, the shear strains in grain interiors become large enough to trigger the deformation twinning.

While the current model did not consider the semi-solid mushy zone explicitly, one may expect that the additional stress generated in this region may further increase the strain inhomogeneity in the solidified microstructure.

## Figures and Tables

**Table 1:** Summary of the elastic constants, twin nucleation barriers, and the stacking fault energies for Al, Ni, and Cu.

Material	$C_{11}[GPa]$	$C_{12}[GPa]$	$C_{44}[GPa]$	$\gamma_{ut}[mJ/m^{2}]$	$2\gamma_{tsf}[mJ/m^{2}]$
Al	114	62	32	215	113
Ni	261	151	132	324	110
Cu	225	153	115	200	40

**Table 2:** Summary of dimensionless parameters for Al, Ni, and Cu.

Material	$C_{11}^{*}$	$C_{12}^{*}$	$C_{44}^{*}$	$\kappa_{11}^{*}$	$\kappa_{22}^{*}$
Al	114	62	32	112	0.09
Ni	121.96	70.56	61.68	49.6	0.09
Cu	140	95.625	71.875	8.77	0.09

**Table 3:** Summary of the critical strain magnitude α and the corresponding critical shear strain magnitude in the reference frame for Al, Ni, and Cu.

Material	α	Critical Shear
Al	0.016	0.0113
Ni	0.006	0.0042
Cu	0.029	0.0205

## Data Availability

The computer codes required to reproduce these findings or data cannot be shared at this time as they are part of an ongoing study.
